# Insight into the Epidemiology of Leptospirosis: A Review of *Leptospira* Isolations from “Unconventional” Hosts

**DOI:** 10.3390/ani11010191

**Published:** 2021-01-14

**Authors:** Giovanni Cilia, Fabrizio Bertelloni, Sara Albini, Filippo Fratini

**Affiliations:** Department of Veterinary Sciences, University of Pisa, Viale delle Piagge 2, 56124 Pisa, Italy; giovanni.cilia@vet.unipi.it (G.C.); sara.albini.p@gmail.com (S.A.); filippo.fratini@unipi.it (F.F.)

**Keywords:** leptospirosis, *Leptospira* isolation, wildlife, zoonoses, host-pathogens interactions, public health

## Abstract

**Simple Summary:**

The isolation of *Leptospira* is the most important test to assess infection in animal species. Several animals play a role as maintenance-host for specific serovars and in the last 30 years the incidence of leptospirosis has constantly increased in well-known reservoirs as well as in “unconventional” hosts. The emergence and the identification of *Leptospira* infection in such “unconventional” hosts could be related to several factors including problematic or inaccurate sampling modes during the *Leptospira* isolation, newly identified *Leptospira* strains, underestimated leptospirosis cases and climatic changes causing modifications of ecological niches. The aim of this review was to report the *Leptospira* isolations of the last 60 years from animals that could be considered “unconventional” hosts. Thus, the identification of “unconventional” hosts is crucial because they almost surely play an important role in the epidemiology of *Leptospira* infection.

**Abstract:**

Leptospirosis is a re-emerging worldwide zoonotic disease. Even though the primary serological test for diagnosis and surveying is the microscopic agglutination test (MAT), isolation remains the gold-standard test to detect *Leptospira* infections. The leptospirosis transmission is linked to maintenance and accidental hosts. In the epidemiology of *Leptospira* some serovar are strictly related to specific maintenance hosts; however, in recent years, the bacterium was isolated from an even wider spectrum of species. The aim of this review is to report the isolation of *Leptospira* strains in animals which could be recognized as “unconventional” hosts, analyzing studies from 1960 to 2020 that highlighted the *Leptospira* isolation. This scientific literature aimed to provide evidence of infection in several animal species including of the Carnivora, Didelphimorphia, Rodentia, Cetacea, Cingulata, Afrosoricida, Chiroptera and Primate orders, as well as in Reptilia and Amphibia classes. In conclusion, the spreading of *Leptospira* is attention-worthy because the infection could occur in all the animal species ranging in a specific area. Further screening and isolations are needed to collect all necessary data to gain a complete understanding of leptospirosis epidemiology and its modifications.

## 1. Introduction

Leptospirosis is a neglected and re-emerging zoonoses caused by a Gram-negative bacterium belonging to the Spirochaetaes phylum, Leptospiraceae family, *Leptospira genus* [1,2]. These microorganisms appear spiral-shaped, with a diameter of 0.1 µm and a length of 6–20 µm and a pointed end that is typically folded into a characteristic hook shape [1]. *Leptospira* is highly mobile and performs rotational movements around the central axis, translation, undulation, and flexion thanks to two periplasmic axial flagella located under the cell membrane [3]. Although *Leptospira* is a microaerophile, it develops well even in conditions of complete aerobiosis. The optimum temperature for its growth is between 28 °C and 30 °C, although it also grows at 37 °C. The ideal pH range is between 7.2 and 7.4 [2].

Traditional classification divided the genus *Leptospira* in two species: *L. interrogans*, pathogenic strains, and *L. biflexa*, saprophytic strains. On the basis of antigenic agglutination reactions and cross absorption, both species are divided into serovars. There are over 60 serovars belonging to the *L. biflexa* species, while there are more than 200 *Leptospira interrogans* [1,2]. Serovars are identified on the basis of their expression of surface epitopes in the mosaic of lipopolysaccharide antigens (LPS), where the specificity of the latter depends on the composition and orientation of sugars [4]. Traditionally, antigenic correlated serovars were grouped into serogroups, which are very relevant in the epidemiological field [5]. Currently, the *Leptospira* genus has undergone a re-classification on a genomic basis, which resulted in the identification of 13 species in addition to those already existing, reaching a total of 64 identified species [1]. This classification system may be more complicated than the previous one as, within the same species, pathogenic and non-pathogenic serovars are included, and specific serovars can be found within several species. For this reason, in several laboratories, the old classification is still used, mainly for convenience in the serological diagnosis [1]. Serovars, identified on a genomic basis, amount to more than 260 and are classified into pathogenic, intermediate, and saprophytic [6,7]. Pathogenic *Leptospira* are the causative agents of moderate to severe forms of the disease, while intermediate *Leptospira* generally cause less severe infections. On the other hand, saprophytic serovars, commonly present in the environment, are not considered pathogenic and can play a relevant role only when they undergo genetic recombination processes with pathogenic serovars [8,9].

The invasion of *Leptospira* into the body occurs through skin lesions (even of minimal entity), via the mucous membrane (conjunctiva and oral mucosa), and by contact with wet skin or by inhalation [2]. Infiltrated microorganisms invade the bloodstream, causing bacteremia that persists for about 5–7 days [10]. Once a critical number of bacteria has been reached in the blood, the first symptoms related to their trans-endothelial migration appear. The pathogenetic mechanism of *Leptospira* is not yet fully understood, and it is hypothesized that virulence factors, such as toxins, adhesins, and other surface proteins, are expressed [11]. Primary lesions affect the endothelium of small vessels and cause ischemic damage in various organs, including kidneys, liver, lungs, meninges, placenta, and muscles [10]. In certain cases, hemorrhages, jaundice due to the destruction of the hepatic architecture, and, more frequently, thrombocytopenia, may also occur [11]. Tissue damage, although severe, can undergo complete healing or its resolution can leave scar tissue, as is often observed in pig kidneys with the characteristic appearance of “white spots” [2].

The primary serological test used for diagnosis and for conducting surveys is the microscopic agglutination test (MAT), but isolation remains the gold-standard test to detect *Leptospira* infections. MAT is performed using a panel of live antigens, selected on the basis of the main serogroups circulating in the reference area, placed in contact with the serum under examination at an initial dilution of 1:100 and subsequent scaling dilutions. The concentration of the free *Leptospira* must be comparable to the negative control, consisting of the culture diluted 1:2 with physiological solution [2,12]. Subsequently, the positivity of the sample, given by the formation of the antigen–antibody complex that manifests itself through agglutination, is evaluated by observations under a microscope in a dark field [13]. It is possible to increase the sensitivity of the test by using locally isolated *Leptospira* instead of those belonging to the classic reference strains [12]. However, in order to diagnose *Leptospira* infections, isolation is the most specific method, even if it is often impractical and complicated, especially considering its time-consuming aspect [12]. Isolation is performed using a liquid medium, such as Ellinghausen–McCullough–Johnson–Harris (EMJH) or Fletcher media. Usually, to stimulate the *Leptospira* growth, albumin, bovine or rabbit serum, and Tween 80 or 40 is added [14]. To avoid the development of contaminating microorganisms, it is possible to use selective agents, such as 5-fluorouracil, nalidixic acid, fosfomycin, polymyxin, bacitracin, and neomycin [12]. The cultures, once prepared, are incubated at 29–30 °C for at least 12 weeks and preferably 26. Checks must be performed every 7–14 days, by observation under a darkfield microscope, to assess the state of bacterial growth [12]. Recently, a new alternative medium, called Hornsby-Alt-Nally (HAN), has been formulated which seems more effective in supporting the growth of *Leptospira* strains, at both 29 and 37 °C. The HAN medium seem to be optimal to perform the primary isolation of fastidious pathogenic strains directly from infected host tissues, especially for strains belonging to *L. borgpetersenii* species [15]. Leptospirosis is a worldwide diffused disease, occurring in tropical, subtropical, and temperate zones [4,16]. The spread of the disease is favored by a large variety of both wild and domestic animals which can be natural reservoirs of *Leptospira* [1,2,10,17,18,19,20]. Several specimens are asymptomatic *Leptospira* renal carriers, contributing to maintain the infection in the environment by constantly shedding bacteria with urine and developing symptoms only after a long period of incubation [2,21]. The incidental contact with *Leptospira* infected urine by non-adapted animals, the so-called accidental hosts, could cause infections that evolve in the above-mentioned clinical diseases. For this reason, the epidemiology of leptospirosis in a particular ecosystem is related to the close relationship between specific *Leptospira* serovars and specific maintenance hosts [17,21,22]. For example, rodents are reservoirs of Icterohaemorrhagiae and Ballum serogroups [23,24], swine of Pomona and Tarassovi serogroups [25,26,27,28,29], horses of Bratislava serogroups [30,31], bovines and ovine of Sejroe serogroups [32,33]. In the last 30 years, the incidence of leptospirosis has constantly increased in well-known reservoirs as well as in never-before detected animal species [34,35,36]. The *Leptospira* incidence in these atypical hosts has become even higher, reaching several animal species from different classes and orders [37,38,39], including a wide spectrum of avian species (seagulls, doves, ibis, and owl) [40,41], reptiles [40,42] and fishes [43]. The emergence and the identification of *Leptospira* infection in such “unconventional” hosts could be related to: (i) problematic or inaccurate sampling methods to isolate *Leptospira* [12]; (ii) newly identified *Leptospira* strains [44]; (iii) underestimated leptospirosis cases, characterized by a downward trend [36]; (iv) climatic changes modifying ecological niches [16,45]; (v) presence of domestic animals raised in semi-extensive or extensive farms, promoting contact with wild species [16,46]; (vi) *Leptospira* strain antimicrobial resistances [47,48,49].

The epidemiology of *Leptospira* and leptospirosis is strictly related to the presence of susceptible hosts, both maintenance and accidental. Since isolation of viable bacteria represents the highest level of diagnosis and the best way to prove the relation between a pathogen and a host, the aim of this review was to report the *Leptospira* isolations of the last 60 years from animals that could be considered “unconventional” hosts. Animal species that are well recognized *Leptospira* hosts were not taken in consideration. Although in many cases, the data available in the literature are not sufficient to classify these “unconventional” hosts as incidental or maintenance, further knowledge of them could give new insights into the epidemiology of leptospirosis.

## 2. *Leptospira* Isolation on “Unconventional” Host

**Figure 1:**
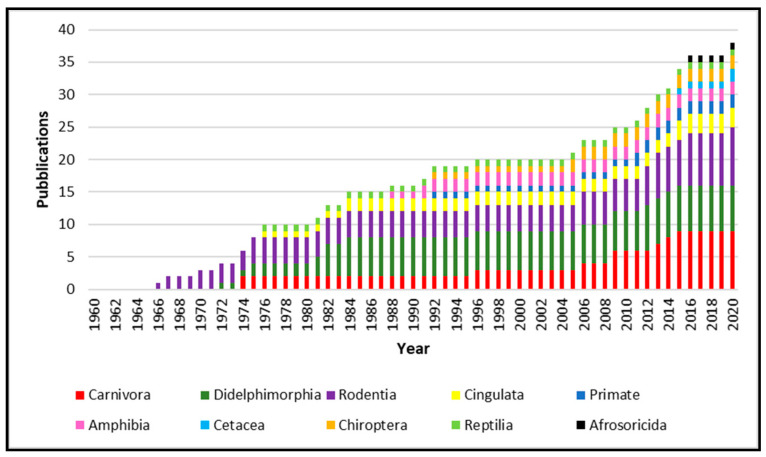
Number of f publications from 1960 to 2020 concerning *Leptospira* isolation from “unconventional” hosts. Different animal orders are represented with different colors.

From 1960 to 2020, 34 papers were published about the isolation of *Leptospira* from species not recognized as leptospirosis reservoir. As showed in Figure 1, the published works increase constantly year after year, reporting *Leptospira* isolation from several animal species. *Leptospira* spp. isolated from animals belonging to the Carnivora order are the most numerous, followed by Didelphimorphia and Rodentia. However, isolation was obtained from Cetacea, Cingulata, Afrosoricida, Chiroptera and Primate, as well as in Reptilia and Amphibia classes.

**Figure 2:**
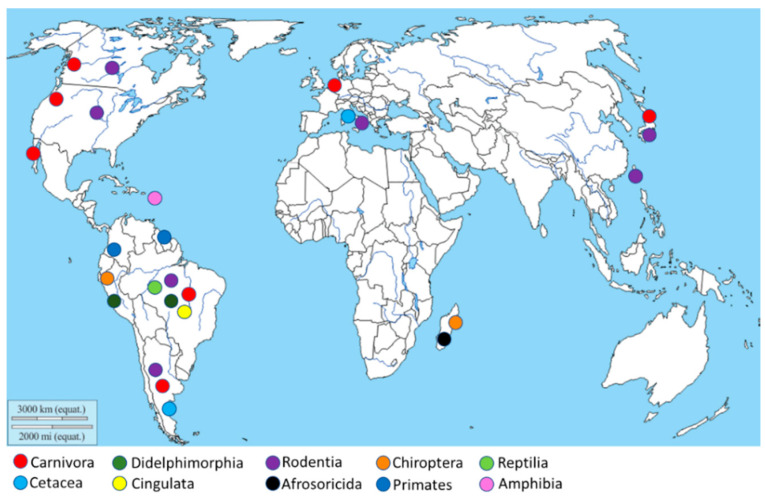
Geographical distribution of the *Leptospira* isolation. The map shows, through different colors, *Leptospira* isolation from different animal orders.

The geographical distribution of *Leptospira* isolation (Figure 2) is more abundant in South America, especially in Brazil and Argentina, due to the high animal species variability present in this geographic area. Moreover, other isolations were performed in the North American West Coast, Italy, Netherlands, Japan, and Madagascar.

### 2.1. Carnivora

Among the Carnivora order, *Leptospira* was isolated from pinnipeds, racoons, and Gray fox (Table 1). Within Carnivora, pinnipeds were the most investigated animals, in which high number of isolations were reported. Within California sea lion (*Zalophus californianus*) and fur seal (*Callorhinus ursinus*) colonies *Leptospira* isolations were obtained from four leptospirosis outbreaks. From all of these, isolations from blood, kidney and urine, *L. interrogans* serogroup Pomona serovar Pomona were obtained [50,51,52,53,54]. The serovar Pomona is considered the most important cause of diseases in California sea lions, as widely reported from serological surveys and post-mortem lesions, reaching the 100% of colony population [50,51,55,56,57,58]. Moreover, serovar Pomona seems to be the reason of high incidence of abortion in this animal species, as suggested by the isolation obtained from its placenta [53]. Considering fur seal, on 29 specimens collected in Alaska, two isolations were obtained from a new-born puppy liver and another one from the urine of an adult male. Both isolations, as for California sea lions, belong to serovar Pomona [53], as demonstrated by MAT surveys on dead animals with hemorrhagic lesions [57]. Moreover, two groups from Phocidae family tested positive for *Leptospira* in the American West Coast, serovar Pomona was isolated from the kidney of a Northern elephant seal (*Mirounga angustirostris*) [59]. However, serological positivity, other than for serovar Pomona, was reported for Grippotyphosa and Bratislava, at high titers [60,61]. Additionally, from a harbor seal (*Phoca vitulina*) sampled in Netherlands, *L. interrogans* serogroup Icterohaemorrhagiae serovar Icterohaemorrhagiae was isolated [62]. The *Leptospira* infection was also detected in these animals through serological and molecular surveys of both free-ranging and captive specimens with even higher positivity, to Icterohaemorrhagiae, Grippotyphosa and Bratislava [60,61,62,63,64,65,66]. From two kidneys of racoons (*Procyon lotor*) *L. interrogans* serogroup Icterohaemorrhagiae and *L. interrogans* serogroup Hebdomadis serovar Hebdomadis were isolated in Japan [67]. This is the first isolation from this animal species, although anti-*Leptospira* antibodies were detected in racoon sera in North America [68,69,70,71]; furthermore, it was demonstrated that, in Canada, trappers contracted leptospirosis from contact with these carnivores [71]. Two isolations were obtained from the Canidae family from South America Gray fox (*Lycalopex griseus*) and Maikong (*Cerdocyon thous*). Serovar Icterohaemorrhagiae was isolated from one out of five south America Gray fox specimens collected in Argentina [72]. True foxes, belonging to *Vulpes* genus, are well recognized as *Leptospira* reservoir, in particular Red fox (*Vulpes vulpes*), [73,74,75], but no isolation was performed among them. *Leptospira* infection was detected only by serological studies that highlighted antibodies against serovars Icterohaemorrhagiae, Canicola, Hebdomadis, Hardjo and Grippotyphosa [76,77]. A *Leptospira* surveillance was never conducted on maikong (*Cerdocyon thous*) until 2015, when in Brazil a strain belonging to Pomona serogroup was isolated from these animals, while Bratislava, Shermani and Whitcombi was also detected by MAT [78].

### 2.2. Cetacea

Among species belonging to the Cetacea order, only two isolations have occurred so far (Table 1). One study describes the characterization of *Leptospira* strain Manara, as collected from a Southern right whale (*Eubalaena australis*) stranded in Patagonia [79]. Moreover, this strain turned out to be halophilic, surviving in a salt environment, at different concentrations, for a few days. The other isolation of serovar Pomona was obtained from the kidney of a common bottlenose dolphin (*Tursiops truncates*) collected in Sardinia (Italy) [80]. These are the only isolations from whale and dolphin species, among several investigated species around the world [40,81,82,83,84,85].

### 2.3. Didelphimorphia

Several opossum species are widely diffused in all South American countries. A very wide diversity of *Leptospira* species have been isolated from kidneys and urines of opossums (Table 1). From the common opossum (*Didelphis marsupialis*) sampled in Peru and Brazil, leptospires belonging to serovar Grippotyphosa, Ballum, Brasiliensis, Szwajzak, Icterohaemorrhagiae, Autumnalis, Tingomaria, Georgia, Huallaga, Rupa rupa were isolated [86,87,88,89,90]. Additionally, in Brazil, *L. borgpetersenii* serovar Castellonis and two strains belonging to serogroup Panama and Pomona, respectively, were isolated from white-eared opossum (*Didelphis albiventris*) [91,92]. Finally, among the Gray four-eyed opossum (*Philander opossum*) in both the Brazilian and Peruvian area, another large variety of *Leptospira* strains were detected [87,89,90,92], as shown in Table 1; in particular, serovars Ballum, Grippotyphosa, Tingomaria, Georgia, and Icterohaemorrhagiae were isolated. Marsupials have been reported to harbor serovars such as *L. kirschneri* serovar Grippotyphosa [10] and *L. interrogans* serovar Canicola [93]. However, all investigated opossum species presented low antibody titers in MAT investigation, suggesting that opossum could be only moderately susceptible to infection [2]. Moreover, opossums experimentally intraperitoneally inoculated with serovar Grippotyphosa did not show clinical signs, although lesions attributed to leptospirosis were observed in liver and kidney tissues [94].

### 2.4. Cingulata

Very few studies were conducted on armadillo’s species and only in Brazil. As reported in Table 1, serogroup Autumnalis, Cynopteri, Hebdomadis and Pomona were isolated from kidneys of nine-banded armadillo (*Dasypus novemcinctus*) [88,95]. Also, a strain belonging to serogroup Pomona was isolated from the urine of a six-banded armadillo (*Euphractus sexcinctus*) [78]. Other serological investigations on armadillo specimens indicate the presence of antibodies to serogroups Autumnalis, Cynopteri, and Pomona at titer reaching 1:1600 [96,97], suggesting that this animal could be susceptible to leptospirosis as an incidental host.

### 2.5. Rodentia

Detailed and recent reviews of literature about leptospirosis in rodent species have been widely treated elsewhere hence they will not be discussed in this article. Indeed, rodents are considered to be one of the most important reservoirs of *Leptospira* [2]. Infections have already been demonstrated in different urban, wild, and imported rodent species, such as *Apodemus, Delomys*, *Mus*, *Necromys*, *Oryzomys*, *Rattus*, *Thaptomys*, *Trinomys* and *Myocastor coypus* [98,99]. However, few data are present in the literature about the role of squirrels and the largest rodents, capybara, and porcupine, in the leptospirosis epidemiology. Among squirrels (Table 1), Ballum and Grippotyphosa serovars were isolated from fox squirrels (*Sciurus niger*) [100,101], while Grippotyphosa serogroup from Southern flying squirrel (*Glaucomys volans*) [102], serogroup Javanica from Pallas’s squirrel (*Callosciurus flavimanus*) [103] and serovar Icterohaemorrhagiae and Canicola from red-bellied tree squirrel (*Callosciurus erythraeus*) [104]. All of these studies highlighted the role of squirrels as renal carriers, although their role in epidemiology remains unknown. The largest rodents in the world, capybara (*Hydrochoerus hydrochaeris*), seem to be incidental hosts of *Leptospira*, as demonstrated by the isolation of serovar Icterohaemorrhagiae and serovar Bananal in Brazil [105,106]. Moreover, other findings suggest that incidental contact with the pathogens indicates asymptomatic infection due to *L. santarosai* identified through molecular amplification [106], and by the experimental infection with a strain belonging to serovar Pomona [107]. Among porcupines, isolations of serovar Pomona were performed only from crested porcupine (*Hystrix cristata*) in Italy, and from North American porcupine (*Erethizon dorsatum*) in Canada [108,109]. The role of this animal species remains unknown because it could be an incidental host or a reservoir of *Leptospira.* However, crested porcupine specimens resulted positive for serogroup Icterohaemorrhagiae (also a titer ≤1:1600), and for Australis and Pomona (at low titers) during a serological survey [110]. Also, anti-*Leptospira* antibodies to serovars Javanica, Hurstbridge, Ballum, Celledoni and Hardjoprajitno were detected [111]. Finally, molecular analysis of the urine of orange-spined hairy dwarf porcupine (*Sphiggurus villosus*) reported a renal *Leptospira* infection, despite the MAT negative results [112], suggesting a role as reservoir.

### 2.6. Afrosoricida

The only investigation on the Afrosoricida order highlighted the isolation of *L. mayottensis* from a tailless tenrec (*Tenrec ecaudatus*) (Table 1) sampled in Mayotte Islands [113]. This valuable piece of information was the nearly perfect identity scored form the tenrec strains and they obtained from ill human patients. The *L. mayottensis* typically circulates in the Mayotte islands, reaching not only humans but also domestic animals [7,50,114]. Probably, tenrec was an incidental host, but a role as reservoir is not to be excluded

### 2.7. Chiroptera

Bats are widely researched about their role in the epidemiology of leptospirosis [123]. However, only a few isolations were performed, highlighting infection by *L. kirschneri* serogroup Grippotyphosa and *L. interrogans* serogroup Icterohaemorrhagiae (Table 1). Strains belonging to serogroup Grippotyphosa were isolated in Peru from the kidney of a striped hairy-nosed bat (*Mimon crenulatum*) and a brown mastiff bat (*Promops nasutus*) [98] and in Madagascar from Seychelles flying fox (*Pteropus seychellensis comorensis*) [97]. Also, serovar Icterohaemorragiae was isolated from the greater spear-nosed bat (*Phyllostomus hastatus*) in Peru [98]. The role of bats in the *Leptospira* spreading remains unknown because all of these isolations seem not to be related to serogroups/serovars detected by MAT [124,125,126,127,128,129], although high prevalence were reported using molecular detection [115,130,131,132,133,134]. Furthermore, increasing the uncertainty of the role of bats as reservoir, several isolation investigations scored negative [115,124], leaving the issue open.

### 2.8. Primates

Within *Leptospira* epidemiology, non-human primates could provide important data due to their close lineage with human beings. Although some investigations were carried out on animals kept in captivity, other studies performed on free-ranging monkeys indicated an interesting prevalence of *Leptospira* infection [135]. The infections occurring in several primate species were mainly caused by strain of *L. interrogans* serogroup Icterohaemorrhagiae and *L. borgpetersenii* serogroup Ballum, serologically detected [136,137,138,139,140,141,142,143,144]. The serological results were confirmed with *Leptospira* isolation in only two cases (Table 1). Among prosimian, blood samples collected from a specimen of ring-tailed lemur (*Lemur catta*) kept in a Portuguese zoological park scored positive to *Leptospira* infection and serovar Copenhageni was isolated [116]. Moreover, serogroup Icterohaemorrhagiae have been isolated from the blood of captive specimens of squirrel monkey (*Saimiri sciures*) in French Guyana and of white-faced capuchin monkey (*Cebus capuchinus* and *Cebus apella*) in Colombia and in Portugal and of a common marmoset (*Callithrix jacchus*) kept in a Portuguese zoological park [116,117,118]. The isolation from squirrel monkey was performed after an outbreak in a colony bred in captivity, where some animals showed jaundice and hemorrhagic syndrome, death, and abortion [117]. All symptoms were referable to leptospirosis and were confirmed by serology and isolation. In the case of white-faced capuchin monkey, too, the isolation was performed after the incoming of leptospirosis symptoms in a colony kept in a recovery center, in which specimens had died despite antibiotic treatments [118].

### 2.9. Reptilia

Among *Leptospira* hosts, reptiles seem to be relevant as well. In these animals, leptospirosis could occur after the ingestion of rodents, typical maintenance-*Leptospira*-host, or due to contact with humid and contaminated environment [42,145]. Moreover, implication and pathogenesis remain unclear in these animals. Presently, only two isolations were reported in two snake species (Table 1). Serovar Andamana strains were isolated in Brazil from a kidney of a Prado’s lancehead snake (*Bothrops pradoi*) [121]. Furthermore, a strain belonging to serogroup Ballum was found in hognosed snake (*Heterodon platghrinus*) [120]. Serogroup Ballum was previously detected in one more snake species [119]. In several snake species, different *Leptospira* serogroups were detected using serological assay [146,147], including Andamana and Ballum [119]. This suggests that snakes are incidental hosts, susceptible to environmental *Leptospira* contaminations due to different landscapes and living fauna.

### 2.10. Amphibia

As for reptiles, little information is available about leptospirosis in toads and frogs. In the Barbados islands, the marine toad (*Bufo maninus*) and the whistling frog (*Eleutherodactylus johnstonei*) were investigated, with very similar results (Table 1). *L. interrogans* serogroup Autumnalis serovar Bim were isolated from three out of four marine toads and two out of three whistling frogs [121,122]. The other specimens were both infected by *L. interrogans* serogroup Australis serovar Bajan [121,122]. Moreover, serological data for toads are available, showing Australis, Autumnalis and Panama infection [122], while no data are present for frogs. No piece of data clarifies if amphibians develop severe and lethal infection, but as well as reptiles, the infections are strictly related to *Leptospira* infection in a specific geographical area. The infection could penetrate in amphibians through water, as these animals can absorb water through their skin, store it in their urinary bladder and reabsorb it during dehydration stress. This mechanism seems to be the most probable cause of leptospirosis in toads and frogs [42].

## 3. Conclusions

Leptospirosis is probably the most prevalent, underestimated, and re-emerging zoonotic disease; animals, both wild and domestic, represent one of its most important transmission sources. This review highlighted several worldwide *Leptospira* isolations from a wide range of a “new” potential reservoir, ranging from rodent, opossum and carnivora species to bats, armadillos, cetaceans, reptiles, and amphibians. The amount of isolation has constantly increased during the last 50 years, suggesting that all free-ranging animals could be an incidental *Leptospira* host, including marine mammals. Probably, *Leptospira* infection is endemic in many countries with no surveillance or diagnostic facilities, especially for animals, such as in several African countries where leptospirosis has rarely or never been reported. These findings are undoubtedly of high importance for human public health, due to the risk of human infection through interaction with the reservoir or incidental hosts or contact with biological materials, including blood, urine, tissue, and excretions. Such risks could affect not only already confirmed worker categories (i.e., veterinarians, trappers, abattoir workers, farm workers, hunters, animal shelter workers and scientists and technologists handling animals in laboratories or during field work) but also marine mammal workers, fishmen, researchers, wildlife rehabilitators, trainers, and zoological park workers.

A better knowledge of the epidemiology of this infectious disease is essential to facilitate the creation of efficient prevention and control programs using a One Health approach. Constant monitoring is needed to control the evolution of the dynamics of leptospirosis epidemiology, mainly focused on new animal species that could contribute to its spreading, in order to better clarify their role as a reservoir or incidental hosts.

## Figures and Tables

**Table 1:** Serogroup and Serovar of *Leptospira* isolated from “unconventional” host specimens, of which are reported Class, Order, Family, Species and Country of collection.

Animal						*Leptospira* Isolation			Year	Reference
Class	Order	Family	Species	Common Name	Country	Samples	Serogroup	Serovar		
Mammalia	Carnivora	Otariidae	*Zalophus californianus*	California Sea Lion	USA	K	Pomona	Pomona	1996	[50]
-	-	-				K	Pomona	Pomona	2009	[115]
-	-	-				U	Pomona	Pomona	1974	[52]
-	-	-				U	Pomona	Pomona	2009	[115]
-	-	-				P	Pomona	-	1974	[53]
-	-	-	*Callorhinus ursinus*	Fur Seal	USA	U	Pomona	Pomona	1974	[52]
-	-	-				L	Pomona	Pomona	1974	[52]
-	-	Phocidae	*Phoca vitulina*	Harbor Seal	NL	B	Ict	Ict	2006	[61]
-	-	-	*Mirounga angustirostris*	Northern Elephant Seal	USA	K	Pomona	Pomona	2014	[58]
-	-	Procyonidae	*Procyon lotor*	Raccoon	JPN	K	Ict	Cop/Ict	2009	[66]
-	-	-				K	Hebdomadis	Hebdomadis	2009	[66]
-	-	Canidae	*Lycalopex griseus*	South American Gray Fox	ARG	K	Ict	Ict	2013	[71]
-	-	-	*Cerdocyon thous*	Maikong	BRA	K	Pomona	-	2015	[77]
-	Cetacea	Balaenidae	*Eubalaena australia*	Southern Right Whale	ARG	K	-	Manara	2015	[78]
-	-	Delphinidae	*Tursiops truncatus*	Common Bottlenose Dolphin	ITA	K	Pomona	Pomona	2020	[79]
-	Didelphimorphia	Didelphidae	*Didelphis marsupialis*	Common Opossum	BRA	K	Bataviae	Brasiliensis	1972	[85]
-	-	-				K	Grip	Grip	1975	[86]
-	-	-				K	Sejroe	Ballum	1984	[87]
-	-	-				K	Mini	Szwajzak	2975	[86]
-	-	-				K	Ict	Ict	1975	[86]
-	-	-			PER	K	Autumnalis	Autumnalis	1981	[89]
-	-	-				K	Cynopteri	Tingomaria	1982	[88,89]
-	-	-				K	Hebdomadis	Georgia	1091	[89]
-	-	-				K	Djasiman	Huallaga	1984	[88]
-	-	-				K	Sejroe	Rupa rupa	1984	[88]
-	-	-	*Didelphis albiventris*	White-Eared Opossum	BRA	U	Borg	Castellonis	2012	[90]
-	-	-				K	Panama	-	1981	[91]
-	-	-				K	Pomona	-	1981	[91]
-	-	-	*Philander opossum*	Gray Four-Eyed Opossum	BRA	K	Pyrogenes	Guaratuba	1975	[86]
-	-	-				K	Ballum	-	1981	[91]
-	-	-				K	Grip	-	1981	[91]
-	-	-			PER	K	Pomona	Pomona	1981	[89]
-	-	-				K	Cynopteri	Tingomaria	1981	[89]
-	-	-				K	Hebdomadis	Georgia	1981	[89]
-	-	-				K	Tarassovi	Luis	1982	[88,89]
						K	Bataviae	Roja	1984	[88]
						K	Ict	Machiguenga	1984	[88]
-	-	-	-	-	-	-	-	-	-	-
-	Cingulata	Dasypodidae	*Dasypus novemcinctus*	Nine-Banded Armadillo	BRA	K	Autumnalis	-	1984	[87]
-	-	-				K	Cynopteri	-	1976	[94]
-	-	-				K	Hebdomadis	-	1975	[87,94]
-	-	-				K	Pomona	-	1976	[94]
-	-	-	*Euphractus sexcinctus*	Six-Banded Armadillo	BRA	U	Pomona	-	2015	[77]
-	Rodentia	Caviidae	*Hydrochoerus hydrochaeris*	Capybara	BRA	K	Ict	Ict/Cop	2012	[104]
-	-	-				K	Grip	Bananal	2016	[105]
-	-	Sciuridae	*Callosciurus erythraeus*	Red-Bellied Tree Squirrel	ARG	K	Ict	Ict	2013	[103]
-	-	-				K	Canicola	Canicola	2013	[103]
-	-	-	*Glaucomys volans*	Southern Flying Squirrel	JPN	K	Grip	-	2006	[101]
-	-	-	*Callosciurus flavimanus*	Pallas’s Squirrel	TWN	K	Javanica	-	2007	[102]
-	-	-	*Sciurus niger*	Fox squirrel	USA	K	Ballum	-	1975	[99]
-	-	-					Grip	-	1967	[99,100]
-	-	Erethizontidae	*Erethizon dorsatum*	North American Porcupine	CAN	B	Pomona	Pomona	1966	[108]
-	-	-				U	Pomona	Pomona	1966	[108]
-	-	Hystricidae	*Hystrix crsitata*	Crested Porcupine	ITA	K	Pomona	Pomona	2020	[107]
-	Afrosoricida	Tenrecidae	*Tenrec ecaudatus*	Tailless Tenrec	MD	K	Mayottensis	-	2016	[112]
-	Chiroptera	Pteropodidae	*Pteropus seychellensis comorensis*	Seychelles Flying Fox	MD	K	Grip	-	2016	[112]
-	-	-	*Phyllostomus hastatus*	Greater Spear-Nosed Bat	PER	K	Ict	Ict	2005	[115]
-	-	-	*Mimon crenulatum*	Striped Hairy-Nosed Bat	PER	K	Grip	Grip	2005	[115]
-	-	-	*Promops nasutus;*	Brown Mastiff Bat	PER	K	Grip	Grip	2005	[115]
-	Primates	Lemuridae	*Lemur catta*	Ring-tailed Lemur	PT	B	Ict	Cop	2019	[116]
-	-	Cebidae	*Callithrix jacchius*	Common Marmoset	PT	B	Ict	Cop	2019	[116]
-	-	-	*Saimiri sciureus*	Squirrel Monkey	FG	B	Ict	Cop	1992	[117]
-	-	-	*Cebus capuchinus*	White-Faced Capuchin Monkeys	COL	B	Ict	Cop/Ict	2011	[118]
-	-	-	*Cebus apella*	Tufted Capuchin Monkeys	PT	B	Ict	Cop/Ict	2011	[116,118]
Reptilia	Squamata	Viperidae	*Bothrops pradoi*	Prado’s Lancehead Snake	BRA	K	Andaman	Andamana	1976	[119]
-	-	Colubridae	*Heterodon platghrinus*	Hognosed Snake	USA	K	Ballum	-	1961	[120]
Amphibia	Anura	Bufonidae	*Bufo marinus*	Marine Toad	BRB	K	Australis	Bim	1991	[121]
-	-	-				K	Australis	Bajan	1991	[121]
-	-	-				B	Autumnalis	Bim	1988	[122]
-	-	-				U	Autumnalis	Bim	1988	[122]
-	-	Eleutherodactylidae	*Eleutherodactylus johnstonei*	Whistling Frog	BRB	K	Autumnalis	Bim	1991	[121]
-	-	-				K	Autumnalis	Bim	1991	[121]
-	-	-				K	Australis	Bajan	1991	[121]

Legend: USA: United State of America; NL.: Netherlands; JPN.: Japan; ARG: Argentina; BRA: Brazil; TWN: Taiwan; CAN: Canada; ITA: Italy; PER: Peru; MD: Madagascar; FG: French Guyana; COL: Colombia; PT: Portugal; BRB: Barbados; K: Kidney; U: Urine; P: Placenta; L: Liver; B: Blood; Ict: Icterohaemorrhagiae; Cop: Copenhageni; Grip: Grippotyphosa; Borg: Borgpetersen.
